# CellPhenoX: An Explainable Machine Learning Method for Identifying Cell Phenotypes To Predict Clinical Outcomes from Single‐Cell Multi‐Omics

**DOI:** 10.1002/advs.202503289

**Published:** 2025-09-23

**Authors:** Jade Young, Jun Inamo, Zachary Caterer, Revanth Krishna, Fan Zhang

**Affiliations:** ^1^ Department of Biomedical Informatics University of Colorado School of Medicine Aurora CO USA; ^2^ Department of Medicine Rheumatology University of Colorado School of Medicine Aurora CO USA; ^3^ Interdisciplinary Quantitative Biology PhD Program BioFrontiers Institute University of Colorado Boulder CO USA

**Keywords:** clinical association, differential abundance, explainable machine learning, Single‐cell multi‐omics

## Abstract

Single‐cell technologies have transformed the understanding of disease heterogeneity, but linking cell‐level phenotypic alterations to clinical outcomes becomes increasingly challenging as single‐cell datasets continue to expand. This is further complicated by the lack of interpretability in existing methods and the difficulty of detecting interaction effects—nonlinear dependencies between factors like sex, age, and disease. To address this, a novel explainable machine learning method, CellPhenoX, is developed to identify cell‐specific phenotypes and interaction effects linked to clinical outcomes. CellPhenoX integrates classification models, explainable artificial intelligence (AI) techniques, and a statistical framework to generate interpretable, cell‐specific scores to uncover condition‐associated cell populations. Extensive benchmarking and applications demonstrate the efficacy of CellPhenoX across diverse single‐cell study designs, including the dedicated and disease‐motivated simulations, binary disease‐control comparisons, and severity‐stratified patient cohorts. Notably, CellPhenoX identifies an activated monocyte phenotype in COVID‐19, with expansion correlated with disease severity after adjusting for covariates and interactive effects. It also uncovers a fibroblast‐specific state transition gradient predicting tissue inflammation in chronic diseases, and identifies therapy‐induced T cell changes and biomarkers linked to the tumor microenvironment. By integrating interpretability into clinical classification, CellPhenoX offers a powerful framework for translating single‐cell findings into clinical impact.

## Graphical abstract

CellPhenoX is an explainable machine learning framework that identifies cell‐specific phenotypes and interaction effects from single‐cell omics data. By leveraging interpretable models, it enables robust discovery of cell‐level phenotypes that contribute to clinical outcomes. Benchmarking on both simulated and real single‐cell datasets, CellPhenoX uncovered critical immune and stromal cell alterations associated with COVID‐19, chronic inflammation, and cancer therapy response, bridging the gap between single‐cell heterogeneity and clinically meaningful insights.

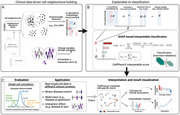

## 1 Introduction

Single‐cell omics technologies have revolutionized our understanding of biological heterogeneity, and offer an opportunity to identify novel cell phenotypes potentially linked to disease pathogenesis.^[^ ^1^ ^]^ With the exponential growth of single‐cell data generating billions of cell profiles through collaborative consortia efforts to unravel biological complexity,^[^ ^2^ ^]^ a new computational challenge has emerged: how to reliably detect and interpret phenotypic changes associated with clinically relevant attributes.^[^ ^3^ ^]^ Early approaches relied on clustering‐based methods for differential abundance testing,^[^ ^4^, ^5^ ^]^ but recent advances have evolved to address this association problem in a cluster‐free manner that provides finer granularity in identifying outcome‐associated cells.^[^ ^6^ ^]^ Most of the cluster‐free methods, such as Meld,^[^ ^7^ ^]^ Milo,^[^ ^8^ ^]^ and Co‐varying Neighborhood Analysis (CNA)^[^ ^9^ ^]^ construct transcriptional neighborhoods to identify cell neighbors associated with sample‐level outcomes. A benchmarking study further demonstrated that Milo effectively maintained accuracy, particularly in the presence of substantial batch effects, while CNA provided scalability for large single‐cell datasets.^[^ ^6^ ^]^ However, these methods primarily rely on linear mixed‐effects modeling, limiting their ability to capture nonlinear relationships and complex interaction dependencies that are fundamental to biological systems. More critically, they lack an interpretable machine learning framework and predictive capability, making them unable to identify predictive and clinically relevant cell phenotypes that could have a transformative impact on diseases.

Given the complexity of large single‐cell data in a multi‐sample, multi‐disease stage, and complex clinical setting, machine learning techniques have the potential to excel in uncovering hidden patterns that traditional statistical models may overlook. However, a fundamental barrier to the adoption of machine learning and artificial intelligence (AI) in single‐cell research is the lack of interpretability. Without a clear understanding of how models arrive at predictions, translating AI‐derived insights into meaningful biological and clinical knowledge remains a challenge. Explainable AI (XAI) strives to provide transparency into model decision‐making and reveal both linear and nonlinear interactions that drive model output.^[^ ^10^ ^]^ Techniques such as the SHapley Additive exPlanations (SHAP) values provide clarity on how features influence model predictions,^[^ ^11^, ^12^ ^]^ which have been applied to identify protein markers for sample‐level measurements in plasma proteomics and bulk experiments.^[^ ^13^ ^]^ However, it remains unclear whether such XAI techniques are capable of identifying cell‐level phenotypic changes that have classifiable and predictable attributes, and whether they are effective for complex disease contexts that often contain confounding factors, such as infectious and immune‐mediated diseases.^[^ ^14^, ^15^, ^16^, ^17^ ^]^

Here, we introduce CellPhenoX, an explainable machine learning tool to identify cell‐specific phenotypes that influence clinical outcomes of interest for clinical single‐cell studies. CellPhenoX classifies clinical phenotypes by integrating machine learning with XAI to generate cell‐specific interpretable scores, providing a quantitative measure of how individual cells contribute to clinical outcomes. Unlike existing models, CellPhenoX directly incorporates cell phenotypes, covariates, and interaction effects into interpretable machine learning, ensuring that clinically relevant associations are detected rather than confounded. Through extensive benchmarking on simulated and real single‐cell disease datasets against other existing methods, CellPhenoX is demonstrated to be robust in detecting condition‐associated cell populations, especially rare cell phenotypes and those influenced by interaction effects, in both binary disease‐control and severity‐stratified clinical outcomes. Based on the generated interpretable insights, CellPhenoX goes beyond statistical correlation‐based approaches, offering a novel framework for uncovering biologically meaningful cell subphenotypes with direct clinical impact.

## 2 Results

### 2.1 Overview of CellPhenoX

**Figure 1:**
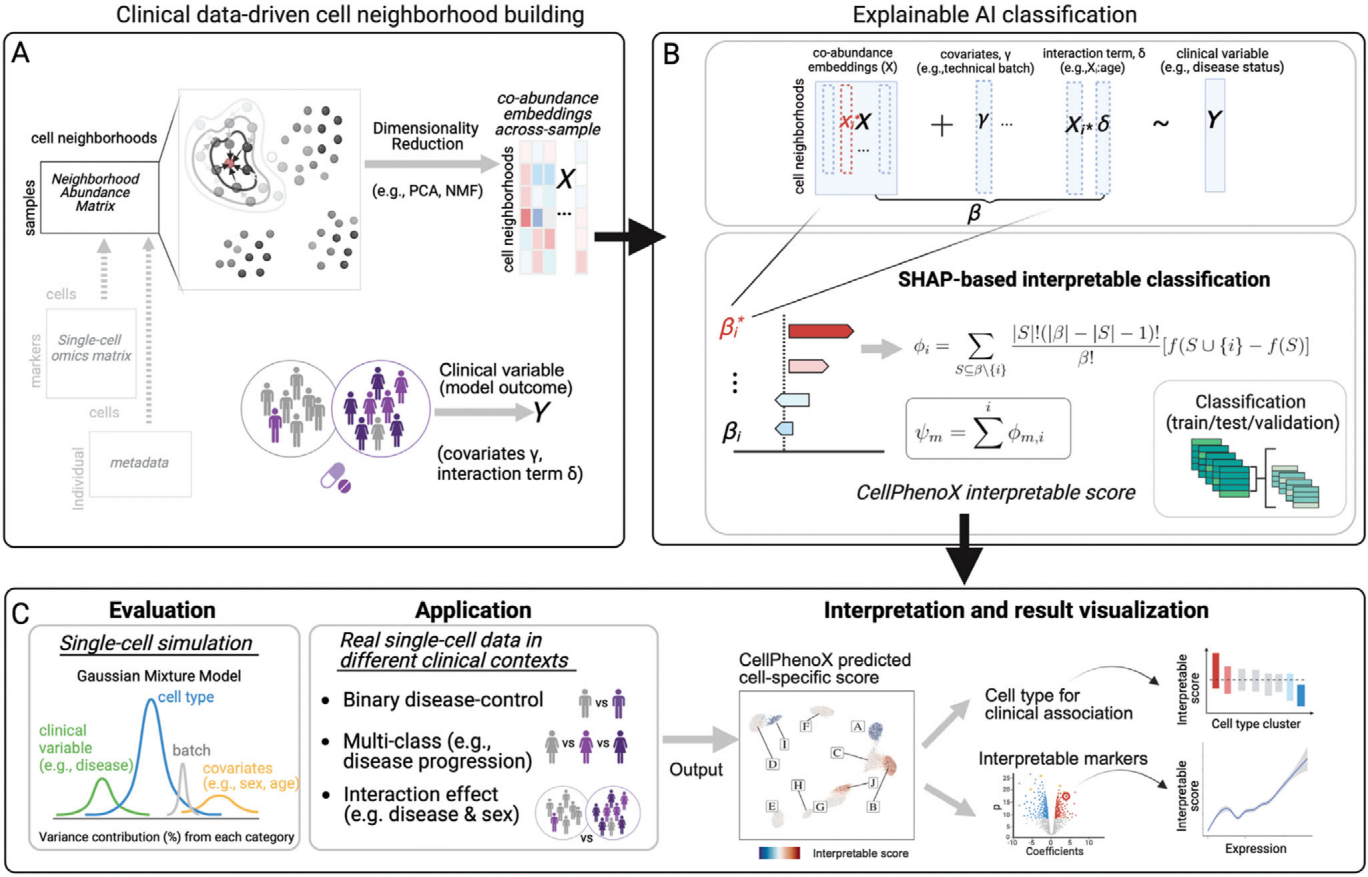
Overview of CellPhenoX methodology. A) CellPhenoX leverages cell neighborhood co‐abundance embeddings, *X*, across samples and clinical variable *Y*, covariates and interaction terms as needed. B) By applying the SHAP‐based explainable AI framework to classification models, CellPhenoX generates interpretable scores, quantifying the contribution of each feature *X_i_*, along with covariates γ and interaction term *X_i_* *δ, to the prediction of a clinically relevant phenotype *Y*. C) CellPhenoX is designed for systemic performance evaluation and applied to diverse, complex single‐cell clinical datasets. Results are visualized at single‐cell level, displaying interpretable scores at low‐dimensional space, annotated cell types for clinical association, and interpretable markers.

The goal of CellPhenoX is to integrate the robustness of classification models with the transparency of XAI methods to produce interpretable, cell‐specific scores that identify cell populations that can be used to classify a clinical phenotype of interest (**Figure** **1**). First, we transform single‐cell gene expression into cell abundance across samples using a neighborhood abundance matrix (NAM), then apply the preferred dimensionality reduction method to the NAM to obtain latent dimensions *X_i_*, where *i* represents the number of latent dimensions (Figure 1A). To predict the clinical phenotype of interest *Y* (e.g., disease status, treatment response), we use *X_i_* as features in our classification models, along with proper covariates γ (e.g., technical batch) and optional interaction effect δ (e.g., age) (Figure 1B). Incorporating these factors explicitly into the model allows us to account for their influence rather than regressing out their effects, thus preserving data integrity and enhancing interpretability. The classification model is trained using a nested cross‐validation (CV) strategy to ensure robustness and prevent overfitting. Final model performance is determined using a hold‐out validation set, providing an unbiased assessment of predictive capability. Within this framework, we employ Shapley Additive Explanation (SHAP)^[^ ^10^ ^]^ values to quantify the contribution of each feature to the prediction for each cell. SHAP provides a local importance score for each feature, allowing us to assess contributions per individual cell. We then rank features based on the distribution of their mean absolute SHAP values, and generate an Interpretable Score by summing the SHAP values across features for each cell (Figure 1B). Next, CellPhenoX is evaluated using our dedicated and disease‐motivated single‐cell simulator, applied on real datasets, and followed by visualization of the interpretable score (Figure 1C). This score identifies and links annotated cell populations that are associated with the clinical phenotype onto a UMAP^[^ ^18^ ^]^ low‐dimensional space. In parallel, markers whose expression distributions are significantly associated with the interpretable score are provided to gain additional biological insights. Since CellPhenoX is designed to model changes in cell abundance based on their predictive or discriminatory capability for clinical metrics, this approach can be generalized to multiple single‐cell sequencing modalities by providing interpretable results that link single‐cell heterogeneity to clinical impact.

### 2.2 CellPhenoX Demonstrates Superior Power on Single‐Cell Simulation Data

**Figure 2:**
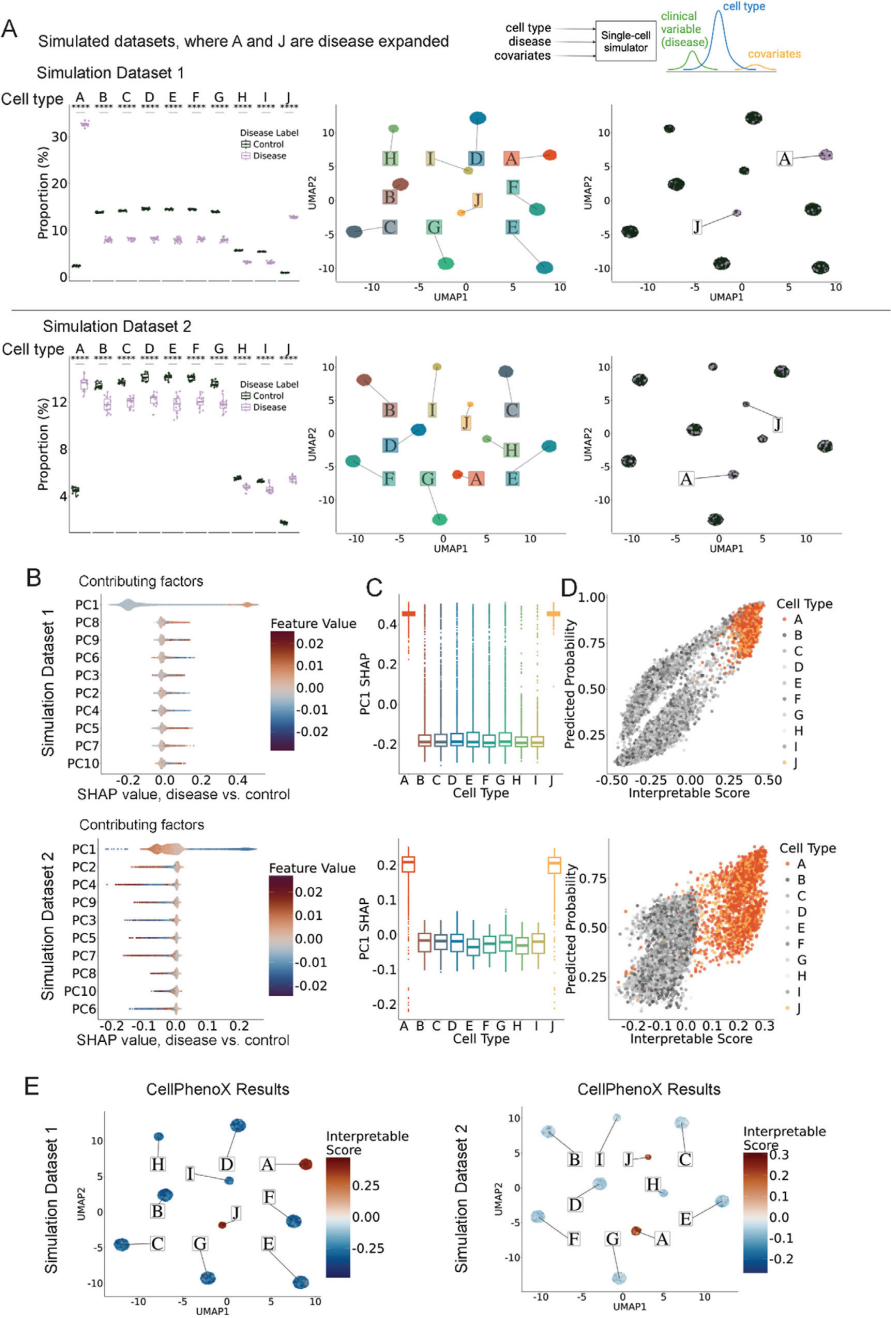
Performance of CellPhenoX on simulation single‐cell datasets. A) Boxplots depicting the proportion of cells in disease and control groups for each simulated cell type cluster, where clusters A and J are disease expanded (left), UMAP plot colored by the cell type cluster (middle) and by disease status (right). The top row represents Simulation Dataset 1, where the fold change between the proportions of disease cells and control cells is 3.0. The bottom row corresponds to Simulation Dataset 2, where the fold change between the proportions of disease cells and control cells is 0.1, B) SHAP summary plots showing the contributing factors ranked by the mean absolute SHAP value where each point represents an individual cell, the color denotes the feature value, and the position along the x‐axis represents the corresponding SHAP value, C) Boxplots showing the distribution of SHAP values across cell types for the most influential feature, PC1 SHAP, D) Scatterplot comparing the discriminatory power of the random forest predicted probabilities and CellphenoX Interpretable Score; here clusters A and J are simulated disease‐associated cell types, E) Visualization of the Interpretable Score indicating disease association on the UMAP plot.

To evaluate the performance of CellPhenoX, we designed our simulated data with varying levels of cell cluster abundance in a disease‐control setting. We simulated two datasets, each with 15 disease samples and 15 control samples, containing ≈100 cells per cell type per sample. Simulation Dataset 1 has 10 cell clusters, with cell type clusters A and J expanded in disease compared to control. Specifically, we made cluster J to represent a rare cell type, which is more challenging to determine in terms of its disease association. In contrast, Simulation Dataset 2 represents a more complex case, where all ten clusters show a slightly higher proportion of disease‐associated cells compared to controls. Compared to controls, the fold change ratio of clusters A and J in diseased samples is 3 in Dataset 1 and 0.1 in Dataset 2, respectively (**Figures** **2A**, and S1A, Supporting Information).

Next, we applied CellPhenoX to each of the simulation datasets by generating a neighborhood abundance matrix (NAM) and decomposing it using PCA. To remove undesirable batch effects and inter‐sample variability, we apply Harmony to these PCs, regressing out experimental factors such as sample or batch. These harmonized latent PCs were input into CellPhenoX to predict disease status using Random Forest (Figure S1B, Supporting Information). Under CellPhenoX, we calculated SHAP summary results highlighting the significant influence of PC1 on the model's predictions (Figure 2B). Notably, PC1 indeed accurately identified clusters A and J as the drivers of the differences between disease and control samples (Figure 2C). Furthermore, we compared the discriminative power of the random forest model's predicted probabilities with CellPhenoX's Interpretable Score. The analysis indicates that our Interpretable Score more effectively separates cell types A and J from the rest of the cell types compared to the random forest model's predicted probabilities (Figure 2D), especially in Simulation Dataset 2. We further presented our CellPhenoX Interpretable Score in the UMAP, confirming that higher scores correspond to cell profiles in the simulated disease expanded clusters A and J (Figure 2E). To assess the generalizability of CellPhenoX, we implemented XGBoost, another widely used tree‐based model, which successfully recapitulated the signal (Figure S2, Supporting Information).

Additionally, we benchmarked CellPhenoX by comparing its performance with CNA^[^ ^9^ ^]^ and MiloR^[^ ^8^ ^]^ evaluating the results from each method against the true disease status of each simulated cell. We found that the median proportion of correct predictions for CellPhenoX across all simulated clusters is 0.848 for Simulation Dataset 1 and 0.710 for Simulation Dataset 2, which outperformed the results from MiloR and CNA (Figure S3, Supporting Information). While MiloR was able to identify cell type A as expanded in disease, it failed to detect the rare cell type J that was also simulated to be expanded (Figure S3C,D, Supporting Information). Additionally, we observed that miloR results may be unstable depending on the selection of cells used to construct neighborhoods (Figure S3E, Supporting Information). Altogether, CellPhenoX demonstrated outstanding performance in identifying condition‐associated cell populations, with superior capability in detecting rare cell populations with precision. While benchmarked methods here reveal statistical correlations relying primarily on linear models, CellPhenoX offers valuable insights into interpretable contribution of individual cells to the predictive outcome.

### 2.3 CellPhenoX Deciphers Interaction Effects Between Sex and Disease

**Figure 3:**
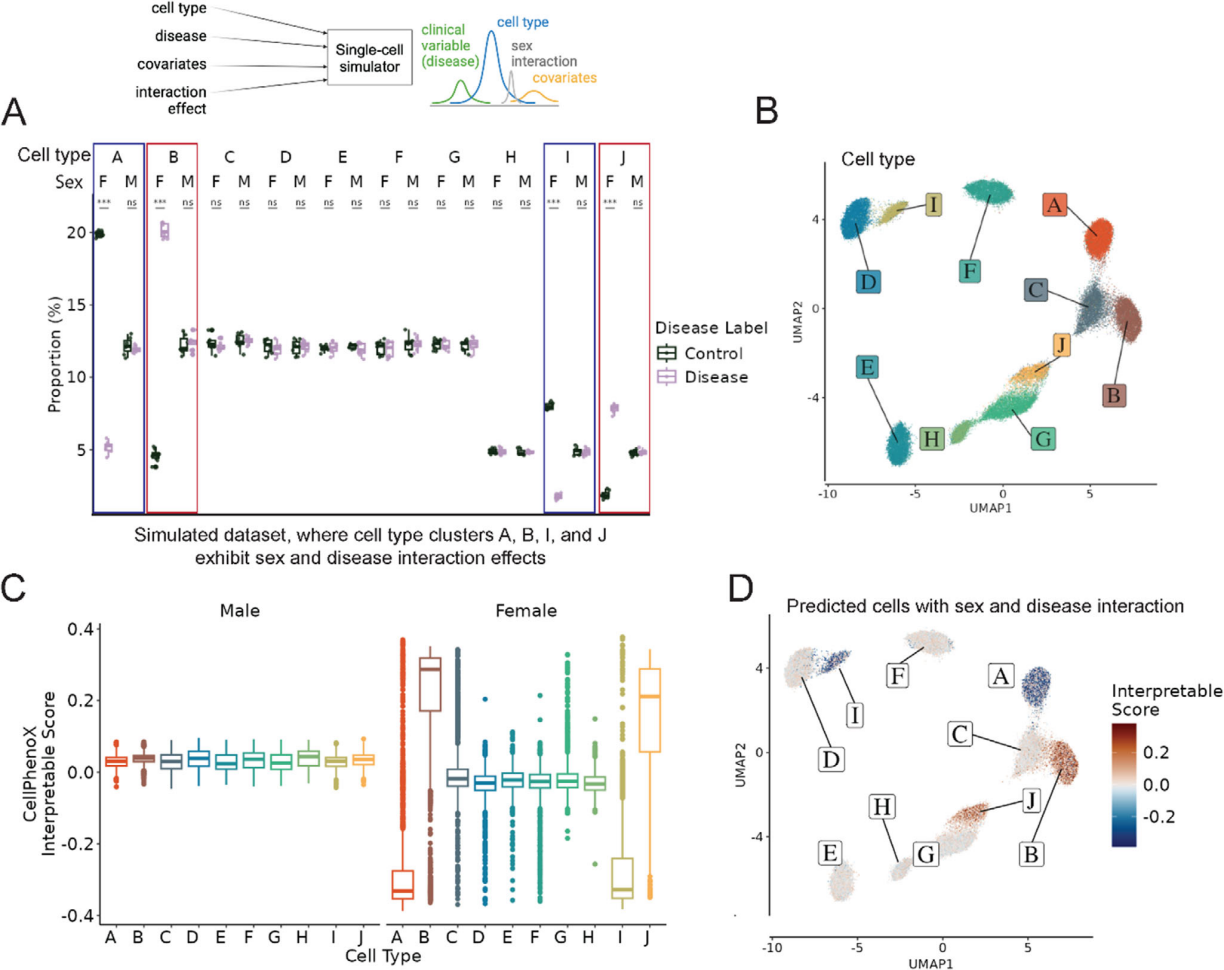
CellPhenoX identifies cell types influenced by disease and sex interactions in simulated single‐cell dataset. A) Boxplots showing the proportion of cells in disease and control groups in Simulation Dataset for each simulated cell type, sex, and disease and sex interaction effect; where “M” is male and “F” is female, B) UMAP plot colored by cell type clusters, C) Boxplots presenting CellPhenoX interpretable scores, faceted by sex, showing score distribution across cell types, D) Visualization of the interpretable scores indicating sex‐predominant disease associations on the UMAP plot.

Disease status rarely manifests in isolation but instead results from an interaction between the condition with other sources of variation, such as sex or age. To our knowledge, there is a need for methods capable of deciphering the interaction effects in single‐cell data, as most existing approaches, such as CNA and MiloR, lack the functionality to detect such interactions. In our study, we simulated a single‐cell dataset featuring differentially abundant clusters influenced by the interaction between sex and disease. This simulation represents a scenario in which disease status interacts with the covariate sex, affecting two major cell types, A and B, and that are depleted and expanded in female disease, respectively (**Figure** **3**A). We also simulated minor cell types I and J, which are also depleted and expanded in female disease, but pose a challenge as they form rare clusters (5%) and also cluster with non‐disease associated cell types in UMAP space (Figure 3A,B). Applying CellPhenoX, we are able to predict binary disease status using the harmonized NAM PCs, their interaction terms with sex, and sex as a covariate. Notably, the most salient feature we identified was the interaction term between the first latent dimension and sex (Figure S4A, Supporting Information). Specifically, we observed that the Interpretable Score was approximately zero for cells from male individuals, while for female individuals, it was ≈0.35 for cell types B and J and ‐0.35 for cell types A and I (Figure 3C). This clearly identified the clusters influenced by the interaction effect. We further demonstrated that the CellPhenoX Interpretable Score recaptured disease‐ and sex‐biased signals in UMAP space, pinpointing both major cell type B and minor cell type J with sex‐biased disease preferences (Figure 3D). Furthermore, we conducted additional benchmarking to assess the extent to which simulated feature interactions influence model performance. We compared the models with and without explicit inclusion of PC:sex interaction terms. When included, latent dimension (LD)_1:sex ranked as the top contributing factor, and the interpretable scores for the simulated expanded cell populations were significantly elevated, highlighting the model's improved ability to capture interaction effects (Figure S4, Supporting Information). These findings highlight that CellPhenoX is capable of extracting fine‐grained disease signals arising from complex interactions between cellular components and covariates.

### 2.4 CellPhenoX Identifies an Activated Macrophage Phenotype in Covid‐19 Using Single‐Cell Proteomics

**Figure 4:**
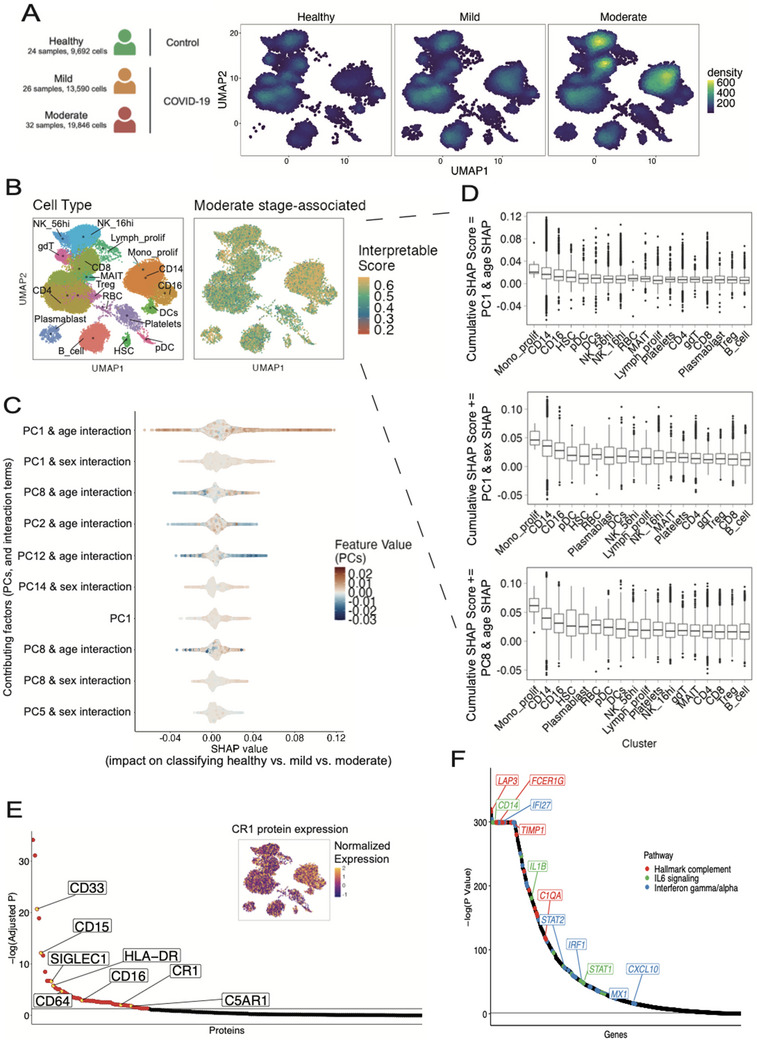
Applying CellPhenoX to single‐cell proteomics of PBMCs from a COVID‐19 study. A) A schematic plot of the multi‐class design including the samples from healthy, mild, and moderate COVID‐19 severity classes (left), and UMAP plots for cell density for each healthy, mild, and moderate classes, B) UMAP plot annotated with the cell cluster labels from the original study (left) and UMAP colored by the Interpretable score by CellPhenoX (right), C) SHAP summary plot showing the top 10 features ranked by the mean absolute SHAP value, where each point represents an individual cell, the color denotes the feature value, and the position along the x‐axis represents the corresponding SHAP value, D) Boxplots showing the distribution of CellPhenoX's Interpretable Score for each cluster starting with the most salient feature (top), then the cumulative SHAP score with the second (middle) and the third (bottom) most influential feature added, E) Proteins sorted by adjusted p‐value regarding their correlations with Interpretable Score. Yellow points highlight specific proteins that are related specifically to monocyte lineage for COVID‐19 severity by literature. CR1 protein normalized expression is presented. F) Genes with expression significantly correlated with interpretable score. Genes are ordered by statistical significance, colored by their inclusion in gene sets representing the three significant inflammatory pathways.

To assess the potential of CellPhenoX to identify specific cell phenotypes implicated in disease, we applied it to a large COVID‐19 proteomics dataset.^[^ ^16^ ^]^ Using CellPhenoX, we performed the multi‐class classification of healthy, mild COVID‐19, and moderate COVID‐19 stages to elucidate cells associated with increasing disease severity. Preliminary investigation of cell density across these conditions revealed an increased abundance of the myeloid cells, NK cells, and CD8+ T cells (**Figure** **4**A). However, this observation is only driven by cell density and does not account for technical effects, sample variation, or key demographic variables such as age and sex. To accurately capture disease‐associated abundance shifts with classification robustness, we are able to predict disease status‐associated abundance changes reflected in the latent components, specifically NAM PCs, adjusting for sex, age, days from disease onset, and smoking status, in our cross‐validation interpretable classification model. Note that the NAM PCs are batch corrected using Harmony^[^ ^19^ ^]^ to reduce the technical batch effect. Furthermore, we included age and sex as interaction terms into our model to capture the complex relationships between these covariates and the latent components. To ensure consistency in providing one Interpretable value for each cell, we aggregated the SHAP values from the three disease statuses as described in the multi‐class SHAP aggregation Methods section. Thus, according to the original cell type annotations^[^ ^16^ ^]^ (Figure 4B left), CellPhenoX's interpretable score highlighted the myeloid cell population as the strongest predictor for the moderate stage (Figure 4B right). The summary plot of the ten most influential latent PCs further underscores the importance of the low‐dimensional cell component interaction terms compared to the standalone cell components, with the interaction of NAM PC1 and age contributing the most to the model prediction (Figure 4C). We further demonstrated the commutative property of SHAP values by decomposing our Interpretable scores and mapping them to cell type annotations. Notably, proliferating monocytes, CD14+, and CD16+ monocytes exhibited a more distinct signal as subsequent features were included in the model (Figure 4D). This conveys the robustness of CellPhenoX's Interpretable Score which consistently identifies proliferating monocytes—a minor but important myeloid cell phenotype for COVID‐19.

The generated Interpretable Score can be used to identify key associated molecular markers. We next examined the proteins with expression levels significantly correlated with the generated Interpretable Score for cells from moderate patients (adjusted *p* < 0.05). In turn, we elucidated key protein markers associated with the discriminative capability of the Interpretable Score, particularly CD33, CD15, SIGLEC1, HLA‐DR, CD64, CR1, C5AR1, and CD16 (Figure 4E). Among these, SIGLEC1 was reported to be expressed by circulating monocytes in COVID‐19 and associated with disease severity^[^ ^20^ ^]^ CD33 was reported as a novel biomarker in peripheral monocytes for COVID‐19 severity^[^ ^21^ ^]^ Further, key immune complement components, including C5AR1 and CR1 (Figure 4E), were uncovered, which were recently shown to increase with COVID‐19 severity leading to the activation of myeloid cells.^[^ ^22^, ^23^ ^]^ Moreover, based on the correlations between gene expression and the interpretable score, we performed gene set enrichment analysis, which revealed significantly enriched pathways such as myeloid cell development and inflammatory pathways, including the complement system, interferon gamma response, and IL6‐JAK‐STAT3 signaling (Figure S5, Supporting Information). Key genes contributing to these pathways include *C1QA*, *LAP3*, *FCER1G*, and *TIMP1* from the complement pathway; *IL1B* and *STAT1* from the IL6 signaling pathway; and *IFI27*, *IRF1*, *CXCL10*, and *MX1* from the interferon response pathway (Figure 4F). Overall, these findings highlight the robustness of CellPhenoX in identifying cell phenotypes and relevant markers associated with changes in disease severity, while accounting for critical covariates and interaction effects.

### 2.5 CellPhenoX Reveals Subtle Fibroblast Changes Associated with Ulcerative Colitis Inflammation and T‐Cell Shifts in Anti‐Pd1 Tumor Treatment

**Figure 5:**
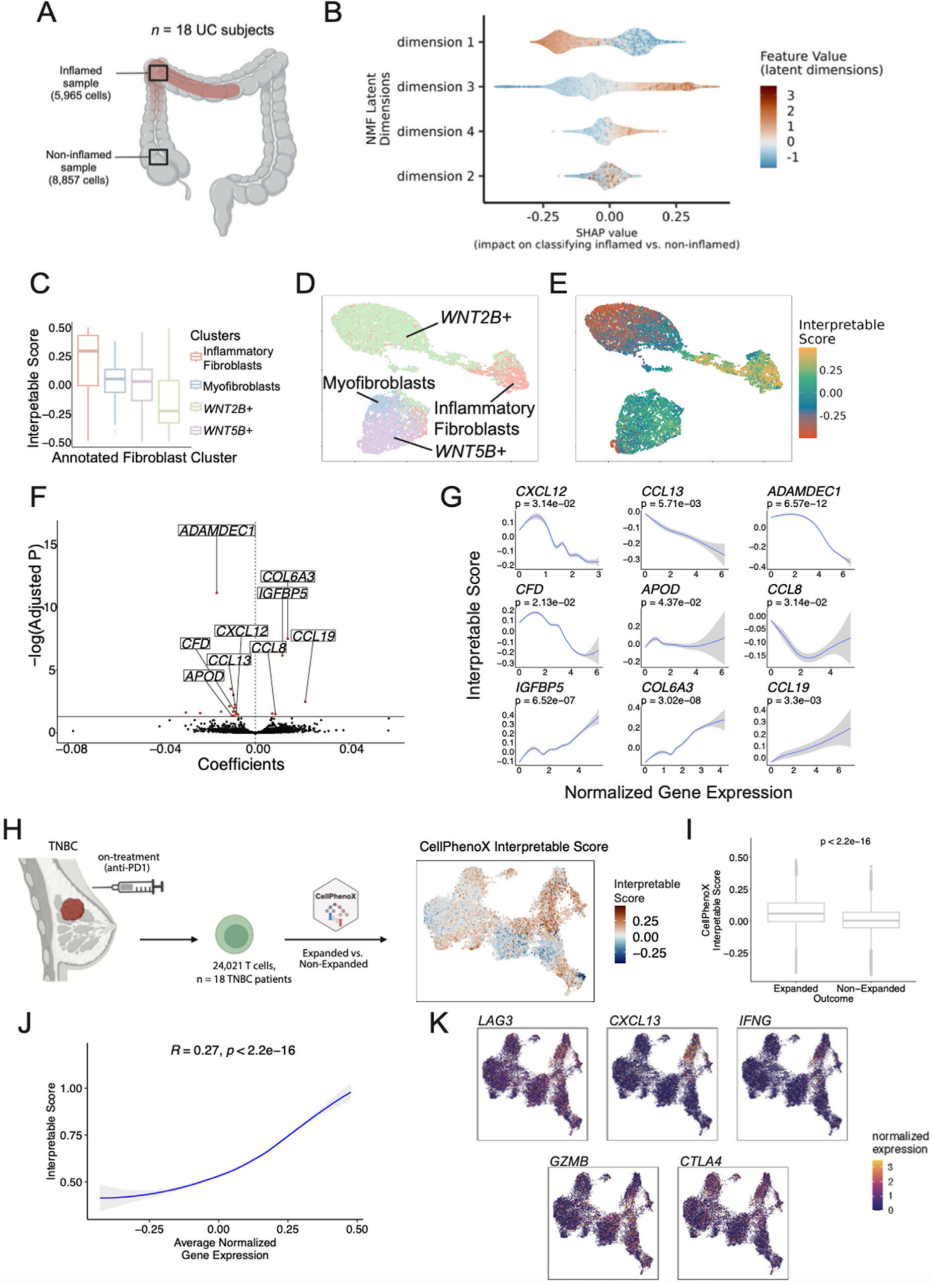
Applying CellPhenoX to single‐cell fibroblast transcriptomics from ulcerative colitis tissue and TNBC datasets. A) Schematic depicting the study design, B) SHAP summary plot showing the NMF latent dimensions ordered by the mean absolute SHAP value, where each point represents an individual cell, the color denotes the feature value, and the position along the x‐axis represents the corresponding SHAP value, C) Boxplots showing the distribution of the CellPhenoX Interpretable Score across fibroblast clusters annotated by the original study, D) UMAP plot colored the annotated fibroblast clusters, E) UMAP plot colored by the CellPhenoX Interpretable Score, F) Volcano plot displaying the correlation coefficients between each gene's expression and the Interpretable Score on the x‐axis, with the adjusted p‐value on the y‐axis. Red points indicate genes with significant correlations, G) Correlation plots of normalized gene expression (x‐axis) and Interpretable Score (y‐axis) for selected significant genes, including 95% confidence intervals and corresponding p‐values. H) Model design for the triple negative breast cancer (TNBC) single‐cell data analysis comparing expanded versus non‐expanded T cells post anti‐PD1 treatment, with UMAP plot colored by CellPhenoX interpretable Score, I) Boxplots of the interpretable score by outcome with Wilcoxon p‐value shown, J) Correlation between the average expression of the top 20 significant genes with the interpretable score, K) UMAP plots showing normalized expression of selected top correlated genes.

It is crucial to identify cell type‐specific variations that are associated with clinical relevance, but this task is often computationally challenging due to the high heterogeneity, especially in diseased tissues. To evaluate CellPhenoX on a specific cell type, we applied it to the single‐cell transcriptomics of fibroblasts from patients with Ulcerative Colitis (UC).^[^ ^24^ ^]^ In this study, adjacent inflamed and non‐inflamed biopsies were collected from patients with UC (n = 18; **Figure** **5**A). To uncover the biological topics underlying cell co‐abundance, we used NMF^[^ ^25^ ^]^ a topic modeling method, and trained a random forest model to predict inflamed versus non‐inflamed status, yielding a classification AUROC of 0.8, AUPRC of 0.86 (Figure S6A, Supporting Information). We observed that NMF‐derived latent dimensions 1 and 3 presented the most influence on the model prediction (Figure 5B). Based on the fibroblast cluster annotations from original study^[^ ^24^ ^]^ we found that the inflammatory fibroblast cluster exhibited the highest Interpretable Score on average (*p* < 2.2e‐16), implicating its critical role in the progression of tissue inflammation (Figure 5C). This is further confirmed by visualizing the cell‐specific Interpretable Score, where inflammatory fibroblasts consistently show the highest scores, with a clear gradient extending toward a subset of *WNT2B+* fibroblasts (Figure 5E). Furthermore, we determined the genes that are correlated with the discriminatory power of the Interpretable Score. Among the 21 significant genes (adjusted *p* < 0.05) (Figure 5F), we identified that *WNT2B+* marker genes (e.g., *ADAMDEC1*, *CXCL12*, *CFD*, and *APOD*) are negatively associated with Interpretable Score, while inflammatory fibroblast marker genes (e.g., *CCL19, CCL8*, *COL6A3*, and *IGFBP5)* are positively associated with the Interpretable Score (Figure 5G). This indicates that the continuous state transition from *WNT2B+* subset to inflammatory fibroblast subset may serve as a key predictor of tissue inflammation, warranting further investigation. In addition, for model robustness, we also tested a different dimensionality reduction method, PCA, which yielded a similar inflammatory fibroblast axis (Figure S6B, Supporting Information). We performed gene set enrichment analysis using MsigDB, based on correlations between gene expression and our interpretable score. The analysis revealed several significantly enriched pathways, including epithelial to mesenchymal transition (EMT) upon growth factor beta (TGFb) stimulation (Figure S6C,D, Supporting Information), as well as histopathological features marked by genes such as *COL6A3*, *IGFBP5*, and *CDH11* (Figure S6E, Supporting Information).

In addition, we applied CellPhenoX on a triple‐negative breast cancer single‐cell data,^[^ ^26^ ^]^ focusing on 24021 T cells, to identify changes between clonally expanded (n = 8) versus non‐expanded (n = 10) after anti‐PD1 treatment divided by clinical outcomes. We presented a CellPhenoX interpretable score for each T cell (Figure 5H), revealing distinct T cell phenotypic differences between expanded and non‐expanded patients. The score effectively separated the two groups (Figure 5I). Next, we presented the top 20 most significant genes that correlated with the CellPhenoX interpretable score (FDR < 0.05, Figure 5J). Among these were *LAG3*, a known marker of T cell exhaustion; *CXCL13*, involved in immune‐cell homing; *IFNG*, an effector T cell marker; *GZMB*, a key marker of cytotoxic function; *CTLA4*, the immune checkpoint marker, all of which were highly expressed by the expanded T cells (Figure 5K). These are well‐established biomarkers associated with T cell expansion following immune‐checkpoint blockade. Together, CellPhenoX is able to decipher therapy‐induced T cell phenotypic changes and highlight potential biomarkers closely linked to the tumor microenvironment.

## 3 Discussion

In this study, CellPhenoX, a novel computational method that integrates machine learning with explainable AI, is introduced to identify cell phenotype shifts that robustly predict clinical phenotypes for single‐cell omics, including transcriptomics, proteomics, and beyond. By leveraging interpretable machine learning, CellPhenoX provides cell‐specific scores that not only distinguish clinical outcomes but also enhance biological interpretability. A key innovation of CellPhenoX is its unique ability to identify cell populations influenced by interaction effects, such as sex and age—an area often overlooked by existing single‐cell methods. For example, CellPhenoX successfully links disease severity with sex and age interactions across simulated, COVID‐19, and tumor single‐cell datasets, respectively. This unique capability positions CellPhenoX as an essential tool for uncovering subtle yet significant interaction effects, shedding light on the intricate mechanisms underlying disease pathogenesis.

Assessing the robustness of single‐cell computational methods in complex clinical datasets is inherently challenging, as the ground truth remains unknown. To address this, we develop a clinically motivated single‐cell simulator capable of generating datasets with controlled variance contributions from disease status, cell type diversity, batch effects, demographic covariates, and interaction effects. This simulation framework serves as a systematic and adaptable benchmarking platform, enabling the flexible modeling of diverse biological scenarios while ensuring a rigorous evaluation of computational methods like CellPhenoX in detecting clinically relevant signals with precision.

Moreover, CellPhenoX's framework applies flexible decomposition methods, including orthogonal and more general matrix decomposition techniques, with adaptable classification algorithms, allowing it to detect subtle and biologically meaningful axes, such as fibroblast differentiation gradients in chronic inflammatory tissues that were difficult to discern. This implementation provides mechanistic insights into cellular behavior while preserving predictive power—a critical advancement to decipher and link heterogeneous cell subphenotypes, like mesenchymal fibroblasts, to clinical outcomes with explainability. Importantly, the design of leveraging methodological versatility and interpretable AI ensures that CellPhenoX can be applied to diverse clinical contexts, showing its ability to capture nuanced cellular behaviors that could inform clinical phenotyping and potential therapeutic targeting.

Despite its strengths, we note several limitations and opportunities for CellPhenoX. First, in scenarios where single‐cell data display high heterogeneity due to clinical variables overlapping with factors like tissue type or cohort difference, the observed cell abundance shifts may introduce potential confounding effects. While CellPhenoX accommodates random and fixed covariates to mitigate these biases, careful contextual evaluation remains crucial to ensure biological validity as it may experience potential overfitting in such complex scenarios. Simulating and detecting higher‐order biological interaction effects, such as nonlinear and multi‐variable covariate effects, remains a major challenge, suggesting the need for continued method development to capture such complex patterns. Second, we may reveal multiple low‐dimensional cell embeddings as interpretable features contributing to the model simultaneously. To maintain the nuance of original characterization, we explain and evaluate the significance of each embedding regarding its contribution to the clinical classifier, and provide a way to aggregate signals to offer global interpretation. Third, applying CellPhenoX on large‐scale single‐cell datasets may be computationally demanding due to the exponential complexity of the Shapley value calculations at the individual cell level. As interpretable machine learning advances, we expect to enhance the scalability of CellPhenoX by adapting it to more computationally efficient implementations in the future. Finally, we utilize tree‐based models like random forests as they are well‐suited for Shapley explanations, maintaining unbiased baseline and marginal Shapley values. We note that deep learning models usually exhibit biases due to the lack of model‐specific algorithms for estimating conditional Shapley values.^[^ ^11^ ^]^ As the theory for explainable deep learning advances, the CellPhenoX framework can be readily extended to incorporate additional classification methods, which we anticipate exploring in future developments.

## 4 Conclusion

In summary, CellPhenoX stands among the first computational tools to directly integrate interpretability with machine learning, uncovering linear, nonlinear, and complex interaction effects in clinical single‐cell datasets, paving the way for translating biological discoveries into meaningful and interpretable clinical impact. While we applied CellPhenoX to single‐cell transcriptomics and proteomics, its design—centered on cell lineages, abundance shifts, and interaction effects—should generalize well to other single‐cell modalities, such as single‐cell ATAC‐seq and spatial transcriptomics. To bridge the gap between algorithmic output and clinical application, CellPhenoX offers several practical avenues for translational use: 1) Single‐cell and patient‐level scoring: Interpretable scores at the cell‐level can be aggregated to quantify clinically relevant phenotypes, including specific immunophenotypic profiles, supporting personalized risk assessment and disease monitoring. 2) Mechanism‐based patient stratification: By capturing cell‐type shifts and interaction effects (e.g., sex‐by‐phenotype), CellPhenoX enables biologically informed subgrouping for precision therapies and clinical trial optimization. 3) Target and biomarker prioritization: Shapley‐based feature attributions highlight genes and pathways most predictive of outcomes, guiding functional validation and therapeutic targeting. 4) Transparent interpretability: The model's explainable outputs enhance clinical trust and facilitate integration into existing decision‐making frameworks. These capabilities position CellPhenoX as a practical and interpretable tool for translating single‐cell insights into meaningful clinical applications.

## 5 Experimental Section

### Datasets—Simulation Overview

Simulating strategy for single‐cell data with differential cluster abundance between conditions

To evaluate the performance of CellPhenoX in identifying associations with clinical variables, a custom, flexible simulation pipeline was developed. This approach generates single‐cell datasets along with corresponding metadata—including disease status, cell type, subject, batch, as well as age and sex when necessary—specifically tailored to evaluate CellPhenoX. The core of the simulation is built based on a Gaussian Mixture Model (GMM) approach, where each feature value represents variance attributable to each factor, including cell type clusters and other sample‐level categorical values. This versatile approach allows control over the source of variation in the dataset through parameters that dictate the proportion of variation explained by each category. The design of the simulation framework relies on the following assumptions.

1) *Distributional assumptions*: The feature values generated for each underlying biological or technical factor, as well as the residual noise, are assumed to follow a Gaussian distribution. The final simulated features, being a linear combination of these Gaussian components, are also implicitly Gaussian.
2) Structure Assumptions: The effects of different biological and technical factors on feature expression are linear and additive.

### Datasets—Simulation Methodology

Metadata was first simulated based on the desired differential abundance. For each differentially abundant cell type *j*, *diff_j_*, the median proportion *p_j_* of *i* in the control group is calculated,

(1) $$diff_{j}=\left[N\cdot \left(p_{j}\cdot \left(1+f\right)-p_{j}\right.\right]-\left[N\cdot p_{j}\cdot f\right]$$

where *N* is the total cell count per subject, and increase in the abundance of cell type *i* in disease subjects by *f*‐fold above the baseline from the control group.

For each simulated feature (e.g., gene or latent representation), the generative process involves the following key steps:

1) Generation of Pure Signals: For each factor *f* ε {*cell* *type*,  *disease*,  *subject*,  *batch*,  *age*,  *sex*}, a pure signal vector was generated $S_{f}\in R^{n}$ for *n* cells. Each element *S*_*f*,*i*_ is drawn from a Gaussian distribution whose mean depends on the level (label) of the factor:
(2) $$S_{f,i}\;∼\;N\left(\mu_{f},\sigma_{f}^{2}\right)$$

where μ_*f*_ is the mean assigned to the cluster (e.g., cluster A vs B). This ensures that the signal is highly specific to the factor it represents. All signal vectors are standardized to zero mean and unit variance across cells.

2) Weighted Linear Combination: Each feature vector, $X\in R^{n}$ is generated as a linear combination of standardized pure signals and noise and scales to have a mean of 0 and a standard deviation of 1. They are then combined using the user‐defined ratio parameters:
(3) $$X=\sum_{f}w_{f}\cdot S_{f}+w_{noise}\cdot S_{noise}$$

where *w_f_* and *w_noise_* are user‐defined non‐negative weights that determine the contribution of each factor and noise to the final feature. The weights are normalized such that:

(4) $$\sum_{f}w_{f}^{2}+w_{noise}^{2}$$

This ensures that the total variance of *X* is 1, allowing precise control over the variance explained (VE) by each component:

(5) $$V\;E_{f}=w_{f}^{2}\;$$

This design enables simulation of scenarios with user‐defined contributions, for example, 40% of variance is due to cell type, 20% due to disease, 10% due to batch, and the remaining 30% is residual noise.

Covariate interaction, such as disease x sex effects could further be modeled through explicit interaction terms:

(6) $$\begin{array}{ccc} X & = & w_{disease}\;\cdot S_{disease}+w_{sex}\cdot S_{sex}+w_{interaction}\cdot \left(S_{disease}\circ S_{sex}\right)\\ & & +w_{noise}\cdot S_{noise} \end{array}$$

To strike a balance between flexibility and control, the simulation framework models interactions between covariates when these are explicitly defined by the user.

### Datasets—Simulation Datasets

For the analyses in this study, the differential abundance of disease‐associated cells with respect to cell type clusters was emphasized. Here, 30 subjects were simulated, each with 100 cells per cell type cluster. Specifically, the simulated dataset included 10 clusters (A through J), where the first seven represented major or more abundant cell types, and the last three were relatively rare cell types. Clusters A and J were designed to be statistically significant expansion in disease compared to control. To achieve differential abundance, the fold change parameter, which controls the magnitude of increase in the number of disease cells relative to control cells was tuned. High fold change values result in more distinct differences in cell proportions between case and control.

For the simulation dataset with desired interaction effect, the cell abundance was further manipulated by introducing a conditional abundance influenced by a covariate (e.g., sex) to simulate a condition‐biased disease status. This simulation framework provides a robust method for evaluating clinically relevant differential abundance and ensures flexibility in generating datasets reflective of varying biological scenarios, such as rare disease‐associated pathogenic cell types.

### Datasets—Real Datasets

1) Ulcerative colitis single‐cell tissue dataset: The Ulcerative colitis (UC) single‐cell transcriptomics dataset includes cells from healthy (n = 12) participants and UC patients (n = 18). Two colon biopsies were collected from UC patients, one from inflamed tissue and one from adjacent non‐inflamed tissue. The analysis was started using their QC‐ed cell matrix, where cluster labels are available driven by their canonical cell lineage marker annotations. Here, the post‐QC fibroblasts obtained from the original study were analyzed, and focused on comparing the inflamed (18 samples, 5965 cells) and non‐inflamed (18 samples, 8857 cells) gut to identify fibroblast‐specific lineages that distinguish these two statuses. The post‐QC count matrix, cell type annotation, and sample‐level metadata were downloaded from link or database ID (Single Cell Portal: SCP259).^[^ ^24^ ^]^
2) COVID‐19 single‐cell PBMC dataset: The COVID‐19 dataset is a single‐cell peripheral blood mononuclear cells (PBMCs) multi‐omics, including surface protein and transcriptome data. Participants in this study ranged the spectrum of disease severity from healthy (24 individuals), hospitalized non‐COVID‐19 (5 individuals), and intravenous lipopolysaccharide (IV‐LPS) (12 individuals) controls, to asymptomatic (12 individuals), mild (26 individuals), moderate (32 individuals), severe (15 individuals), and critical (17 individuals) COVID‐19. The single‐cell count matrix together with classified cell type labels was obtained, where there were 781123 total cells after quality control with manually annotated cell populations based on expression of canonical markers and surface proteins. Clinical data is also available, including days from onset, smoker status, age, sex, status on day of sample collection, and the most severe status that the participant progressed to. For the study of interest, the single‐cell proteomic data from mild (13590 cells, 26 samples), moderate (19846 cells, 32 samples), and healthy control (9692 cells, 24 samples) groups were examined. This dataset is downloaded from the following link: (https://www.ebi.ac.uk/biostudies/arrayexpress/studies/E‐MTAB‐10026).^[^ ^16^ ^]^
3) Triple‐negative Breast Cancer single‐cell tumor dataset: The Breast Cancer dataset is a single‐cell transcriptomic dataset of primary breast tumors^[^ ^26^ ^]^ Participants in this study were diagnosed with one of the three main subtypes of breast cancer: ER‐/HER2+ (2 individuals), ER+/HER2‐/+ (15 individuals), and triple‐negative breast cancer (TNBC) (12 individuals). One cohort of patients were treated with pembrolizumab, an anti‐PD1, and the second cohort received neoadjuvant chemotherapy. Focused was placed on the T cells of the patients with TNBC on pembrolizumab, totaling 24021 cells, by comparing clonally expanded (n = 8) versus non‐expanded (n = 10) after anti‐PD1 treatment. This dataset was downloaded from the following link: (https://drive.google.com/file/d/1ZFte8Cs5k59diuOtj_9W0LngNxTXq99O/view?usp = sharing).

### Data Preprocessing and Dimensionality Reduction

For single‐cell datasets, the raw count matrices were first normalized based on log transformation, followed by selecting highly variable genes based on dispersion. Let this normalized matrix, *Z*, have *M* cells and *P* features. For the *N* samples, *C*(*n*) is denoted as the set of cells that come from sample *n*. Anchoring at each cell, a neighborhood could be defined based on the adjacency suggested by a nearest neighbor graph. In single‐cell transcriptomics data, the neighborhood captures transcriptional similarities, while in single‐cell proteomics data, it reflects proteomic similarities. Therefore, two cells that are in close proximity in the neighborhood can be linked by a random walk in the graph. Then,, a weighted *M* × *M* adjacency matrix A is generated to measure the similarity between cells *m* and *m*′ in the graph. In *A*, a number of steps, *s*, and a binary encoded vector, *e*, where the *m*‐th entry is 1 and 0 otherwise, the probability that a random walk from *m*′ ends at *m* can be defined as:

(7) $$P_{m^{\prime }\to m}^{s}≔{\left(e^{m^{\prime }}\right)}^{T}{\hat{A}}^{S}e^{m}$$

Then, a neighborhood abundance matrix (NAM) was generated^[^ ^9^ ^]^ by first defining:

(8) $$R_{n,m}≔\sum_{m^{\prime }\in C\left(n\right)}P_{m^{\prime }\to m}^{s}$$

where *R*_*n*,*m*_ is the total number of cells from the *n*‐th sample that are expected to be in neighborhood *m*. Now the NAM, *Q*, is determined by normalizing the rows of *R* so that each row sums to 1 as:

(9) $$Q_{n,m}=\frac{R_{n,m}}{\sum_{m}R_{n,m}}\;$$

The aim is to decrease the number of dimensions of the NAM, *Q*, and then use the derived latent dimensions that summarize the major variation as features to prevent overfitting of the classification model in the next step. Herein, CellPhenoX offers the flexibility to employ different dimensionality reduction techniques on the calculated NAM. Thus, users were provided with with the option to choose between two widely used methods for single‐cell analyses—principal component analysis (PCA) and non‐negative matrix factorization (NMF). A notable difference between these two techniques is that PCA learns orthogonal latent dimensions based on the captured variation, whereas NMF, based on topic modeling, does not enforce orthogonality in the learned dimensions. Specifically, for PCA analyses, learning was initiated with 100 components, and then selected the top 20 components that achieved the desired variance explained. For NMF, if the number of ranks (cell clusters) to learn, *k*, is not provided by prior knowledge or the users, a range of *k* values was tested and the one with the highest silhouette score was selected. In the case of the ulcerative colitis single‐cell dataset, this was used as an example to evaluate the performance of both methods, and found that the most influential latent dimensions from both methods summarize similar sources of variation. Considering the event of strong batch effects in single‐cell datasets, Harmony^[^ ^19^ ^]^ was applied with default parameters to remove sample‐specific and technical batch‐associated effects. Harmony is a well established and utilized algorithm for removing batch effects from single‐cell data. Harmony accomplishes this by projecting cells into a shared low dimensional space and iteratively grouping them based on biological similarity such as cell type rather than technical confounders like batch. In the framework, undesirable effects from features such as sample/donor id and batch were removed using Harmony, but retain the variations due to sex and age since these covariates are often directly implicated in disease heterogeneity.

### Classification Model Training and Prediction

After dimensionality reduction, *X_i_* was denoted as the learned latent dimension embeddings, ϒ be the vector(s) of covariates with length *M* (number of cells), δ_*i*_ be the variables that measure the interaction effect with latent embedding *i*, and *Y* be the vector for the target variable (clinical phenotype of interest). Herein, *Y* and ϒ are label encoded if they are categorical variables. The set of all contributing factors, or predictors could be represented, as:

(10) $$\beta =X_{i}+\gamma +X_{i}*\delta_{i}$$

then, the classification model, with θ parameters was designed, to predict $\hat{Y}$ in the following formula:

(11) $$\hat{Y}=f\left(\beta ;\theta \right)$$

For a random forest model with a categorical outcome, $\hat{Y}$ can further be represented as a majority vote from the *T* trees:

(12) $$\;\hat{Y}=mode\left(f_{1}\left(\beta \right),f_{2}\left(\beta \right),\text{…}f_{T}\left(\beta \right)\right)$$

To train the random forest model, the data was split into training, testing, and validation sets using stratified *k*‐fold strategy to maintain the relative class proportions in each fold. The outer loop consists of training and testing, while the inner loop involves training and validation. Predictions are made on the testing data from the outer loop, and final model performance is evaluated on the validation set.

### Hyperparameter Optimization for Classification

To ensure the robustness of the classification model and the resulting SHAP values, a nested cross‐validation procedure that includes both hyperparameter tuning and cross‐validation was used. Selecting appropriate hyperparameters for the dataset and performing cross‐validation to mitigate fold‐split bias are crucial for optimal model performance. Since there are many parameters to tune for random forest, a randomized search across the parameter space was opted for instead of an exhaustive search.

### SHAP Value Calculation

The SHAP value for each feature β_*i*_ is denoted as ϕ_*i*_, representing the contribution of *X_i_* and other involved covariates to the predicted outcome *Y*. This contribution is determined by:

(13) $$\phi_{i}=\sum_{S\subseteq \beta \setminus \left\{i\right\}}\frac{\left|S\right|!\left(\left|\beta \right|-\left|S\right|-1\right)!}{\beta !}\;[f\left(S\cup \left\{i\right\}\;-\;f\left(S\right)\right]$$

where *S* is a subset of features excluding β_*i*_, and $f\left(S\;\cup \;\left\{i\right\}\right)$ and *f*(*S*) are the model predictions with and without β_*i*_, respectively. In Equation (7), all possible subsets of features not containing β_*i*_ are iterated over andthe average marginal contribution of each feature β_*i*_ is calculated across these subsets. The numerator determines the product of the number of ways to order features in subset *S* and the number of ways to order the remaining features, divided by the total number of ways to order all features in β. The average is then multiplied by the difference in model prediction with and without β_*i*_, highlighting the impact of β_*i*_ on the model output.

For implementation, Fast TreeSHAP was integrated into the package by adopting the TreeExplainer function from the fasttreeshap package using the best estimator and the testing set for the outer loop. It was found that Fast TreeSHAP consistently outperformed the shap package TreeExplainer algorithm in computational time, with the performance advantage becoming more pronounced as dataset size increased (Figure S7A, Supporting Information), while retaining accuracy in both methods (Figure S7B, Supporting Information). After completing all CV repeats, each of the cell IDs was iterated over and take the average of all SHAP values for each feature across repeats. The resulting data frame maintains the original input dimensions: *N* cells by *M* features. Let *D^b^* be the data frame containing the SHAP values, where *b* is the number of CV repeats. For each cell *n* the following is calculated:

(14) $${\bar{S}}_{n,j}=\frac{1}{b}\;\sum_{i=1}^{b}S_{n}^{\left(i\right)}$$

where *S* ^(*i*)^ is the *i*‐th SHAP matrix, *S_n_* ^(*i*)^ represents the values in row *n*, and ${\underline{S}}_{n,j}$ is the mean value at row *n* across all *b* matrices. The final matrix of SHAP values was then created as:

(15) $$\bar{S}=\left[\begin{array}{cccc} {\bar{S}}_{1,1} & {\bar{S}}_{1,2} & \text{…} & {\bar{S}}_{1,m}\\ {\bar{S}}_{2,1} & {\bar{S}}_{2,2} & \text{…} & {\bar{S}}_{2,m}\\ \vdots & \vdots & \vdots & \vdots \\ {\bar{S}}_{n,1} & {\bar{S}}_{n,2} & \text{…} & {\bar{S}}_{n,m} \end{array}\right]$$

### Multiclass SHAP Value Aggregation

When dealing with more than two classes, the SHAP values from each class need to be combine to yield one SHAP value per cell per feature. Where there are several ways to do this, this was approached by letting *D* be the matrix of SHAP values with *N* cells and *M* features. For each cell *i*, the SHAP values from the matrix that correspond to the cell's predicted class were selected:

(16) $$\begin{array}{ccc} D_{\mathrm{combined}} & = & \left\{D_{0}\left[i,\;:\right]\;\mathrm{if}\;i\;=0,\;D_{1}\left[i,\;:\right]\right.\\ & & \left.\;\mathrm{if}\;i\;=1,\;\text{…},\;D_{c}\left[i,:\right]\;\mathrm{if}\;i\;=\;c\right\} \end{array}$$

where *c* is the number of classes minus one.

### CellPhenoX Interpretable Score

For each cell *m*, the Interpretable Score, ψ_*m*_, was obtained by summing the obtained SHAP values across feature *i* in ϕ_*m*,*i*_. This score summarizes the contribution of each cell to the overall classification outcome:

(17) $$\psi_{m}=\sum^{i}\phi_{m,i}$$

### Benchmarking Methods and Performance Evaluation—CNA

Co‐varying neighborhood analysis (CNA)^[^ ^9^ ^]^ is built to identify co‐varying cell abundance shifts across involved individuals with respect to clinical outcomes or sample differences in a linear‐based framework. Anchoring at the cell, CNA constructs a nearest neighbor graph describing the transcriptional relatedness of individual cells from each sample. Neighborhoods are determined by quantifying the probability that a random walk from one cell will end at another cell. Then, CNA constructs a neighborhood abundance matrix (NAM), *Q*, a sample by neighborhood matrix, to indicate the relative abundance of neighborhood *m* in sample *n*. Assuming a Gaussian distribution, CNA tests the association with a sample‐level covariate or outcome with a linear regression model:

(18) $$y=U^{k}\;\beta^{k}+\in$$

where *U^k^* denotes the first *k* columns of *Q*’s left singular vectors, *U*, β^*k*^ is the *k*‐length vector of coefficients, and ε is the zero mean Gaussian noise. A multivariate F‐test is employed to determine the P‐value across a range of *k* values. Then, given a false discovery rate threshold, the differentially abundant neighborhoods are identified by computing a smoothed correlation between each neighborhood m and the sample‐level covariate *y*. CNA was run with the default parameters given by the tutorials for the R implementation (version 0.0.99), and controlled for sample‐sample differences. Specifically, the sample was controlled as covariate from the simulation disease‐control single‐cell data, and reported cells whose neighborhood correlation to disease at FDR < 0.05.

### Benchmarking Methods and Performance Evaluation—MiloR

MiloR is a method designed to refine the Cydar^[^ ^4^ ^]^ framework by incorporating neighborhood‐based analysis for differential abundance.^[^ ^8^ ^]^ Milo was chosen for its ability to detect cell population shifts in single‐cell data, addressing the limitations of hypersphere‐based methods such as Cydar. Milo uses a dynamic k‐nearest neighbors (kNN) graph, enabling adaptive and context‐sensitive neighborhood formation. The neighborhood formed for a cell is based on its nearest neighbors using the findKNN() function from the BiocNeighbors package. To detect differential abundance, MiloR applies negative binomial generalized linear models (NB‐GLMs) via the edgeR package^[^ ^27^ ^]^. To optimize computational efficiency, the parameter prop was first set to 0.1, suggested as default by the developers, to identify cell neighborhoods based on a randomly subsampling. Sensitivity analysis was further performed by changing this parameter across 0.1, 0.3, and 0.5. For reproducibility and consistency with established workflows,MiloR was implemented using version 2.1.0 of its R package (https://github.com/MarioniLab/miloR).

## Conflict of Interest

The authors declare no competing interests.

## Author Contributions

F.Z and J.Y. conceptualized the study. J.Y. developed and implemented the algorithm, and generated the results and figures. J.I. implemented the single‐cell simulation pipeline. R.K. and Z.T.C. finalized the software package. J.Y. and F.Z wrote the manuscript with input from the remaining authors.

## Supporting information



Supporting Information

## Data Availability

The original data used in this paper can be accessed at the following repositories with details described in the Experimental Section: 1. Ulcerative Colitis single‐cell tissue dataset (Single Cell Portal: SCP259), 2. COVID‐19 single‐cell PBMC dataset (https://www.ebi.ac.uk/biostudies/arrayexpress/studies/E‐MTAB‐10026), 3. TNBC single‐cell tumor dataset (https://drive.google.com/file/d/1ZFte8Cs5k59diuOtj_9W0LngNxTXq99O/view?usp = sharing). The CellPhenoX software and source code are available at https://github.com/fanzhanglab/pyCellPhenoX. The single‐cell data simulation code is available at https://github.com/fanzhanglab/SCORPIO.
