# Supplementary material for: CellPhenoX: An Explainable Machine Learning Method for Identifying Cell Phenotypes To Predict Clinical Outcomes from Single‐Cell Multi‐Omics
Source: Adv Sci (Weinh). 2025 Sep 23;12(42):e03289. doi: 10.1002/advs.202503289 (PMC12622556; doi:10.1002/advs.202503289)
Supplement: Supplementary file 1 — Supporting Information [file ADVS-12-e03289-s001.pdf]

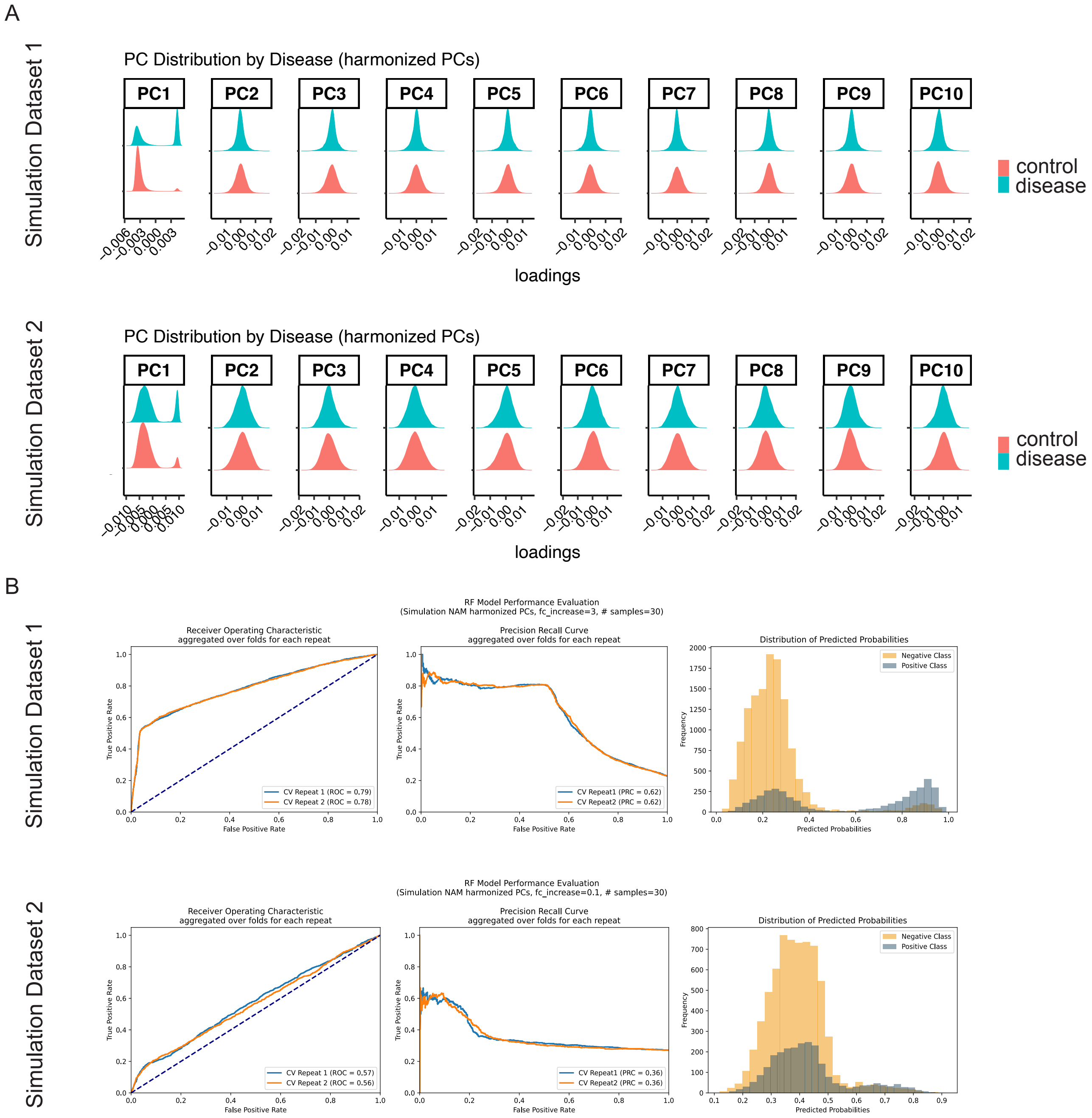

Supplementary Figure 1. The simulation schema produces differential abundance signals that influence the model performance. A. Density plots reveal the changes in distribution of the PC loadings by disease status for simulation dataset 1 (top) and simulation dataset 2 (bottom), **B.** Random Forest model performance for simulation dataset 1 (top) and 2 (bottom), including the AUROC curves for different CV repeats (left), the AUPRC curves (middle), and histograms showing the predicted probabilities for the positive (disease) and negative (control) classes.

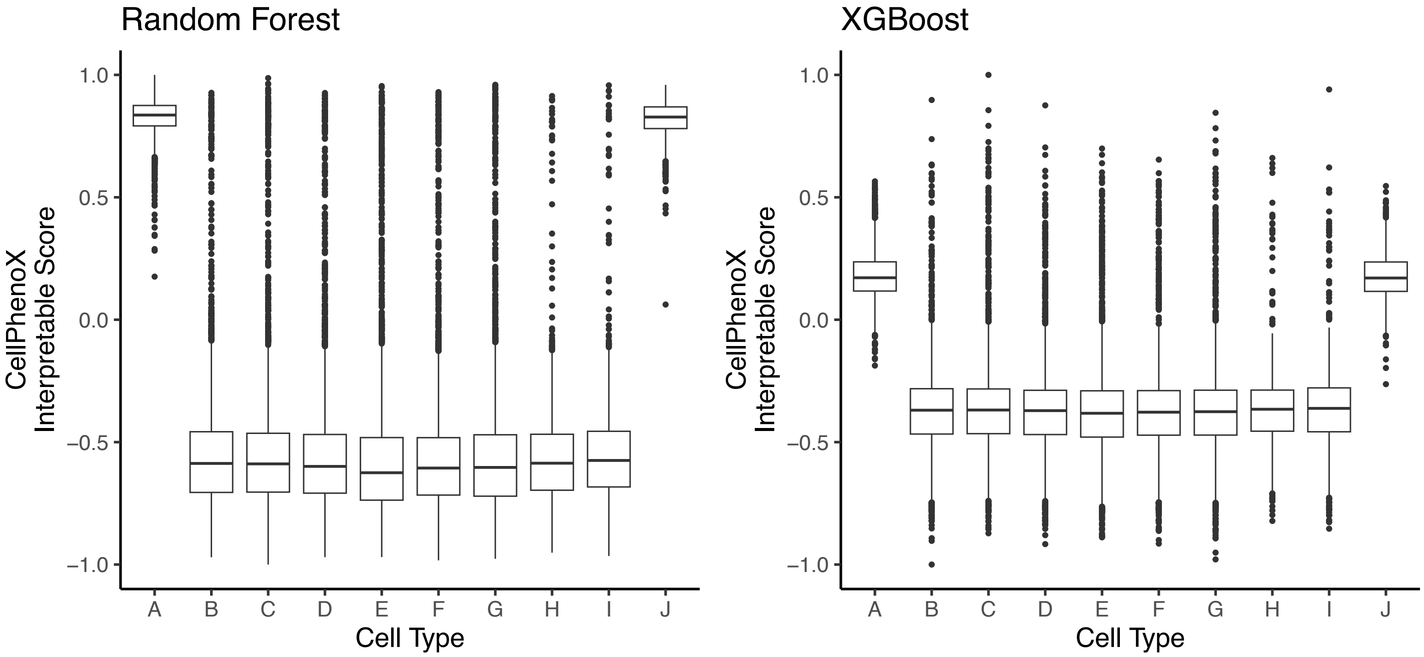

Supplementary Figure 2. Comparison of CellPhenoX between Random Forest and XGBoost on simulational datasets.

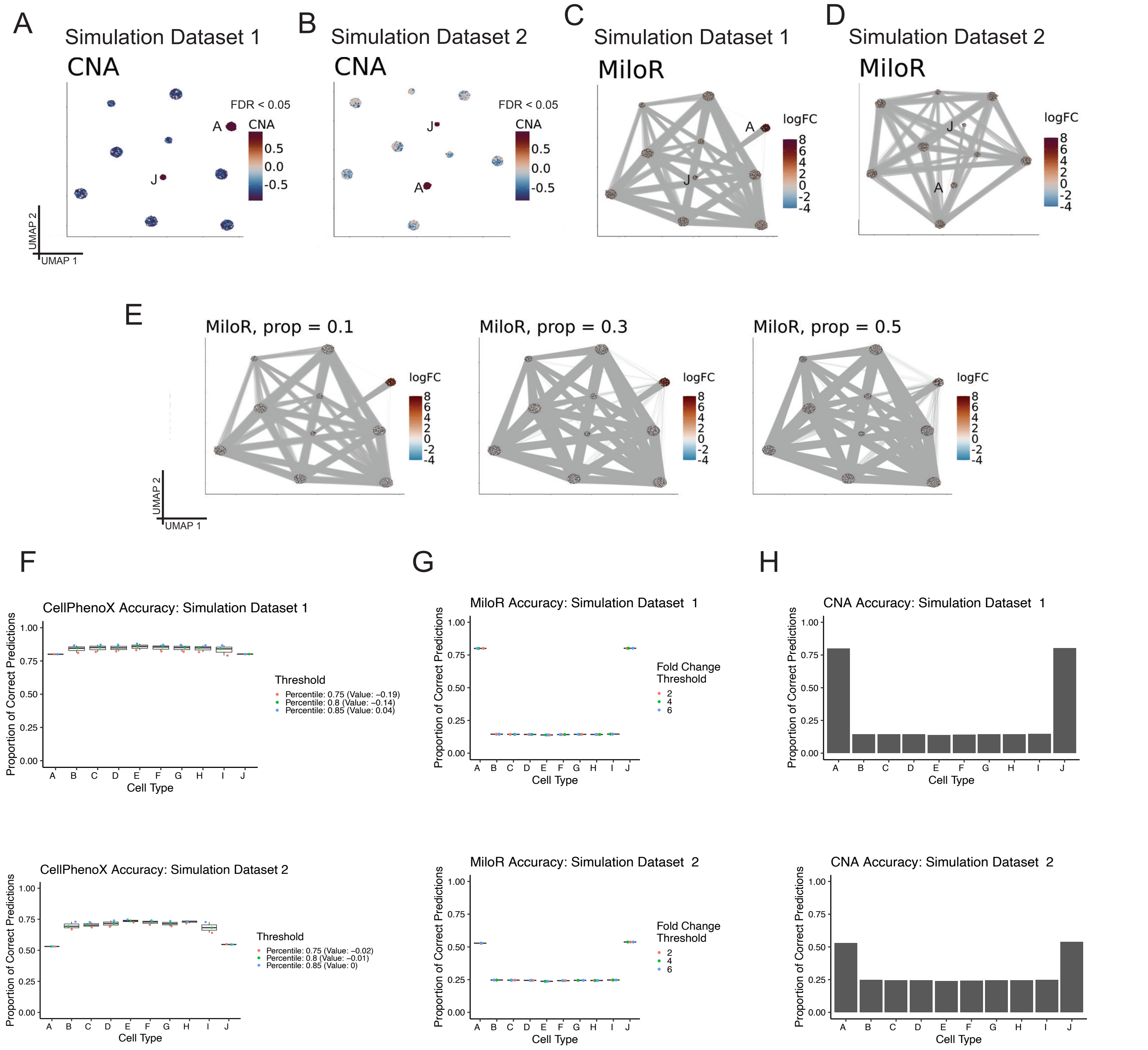

Supplementary Figure 3. Details of benchmarking with CNA and MiloR using single-cell simulation datasets. **A.** UMAP colored by CNA local correlation values (FDR <0.05) for disease for simulation dataset 1, **B.** UMAP colored by CNA local correlation values (FDR <0.05) for simulation dataset 2. Note that cells no passing FDR<0.05 are shown in white, **C.** UMAP colored by MiloR log fold change for simulation dataset 1, **D.** UMAP colored by MiloR log fold change for simulation dataset 2, **E.** UMAP colored by MiloR log fold change by varying the sample proportion parameter value, prop=0.1 (left), prop=0.3 (middle), prop=0.5 (right) (simulation dataset 1), **F.** Boxplots depicting the proportion of correct predictions by CellPhenoX for each simulated cluster. Different thresholds for binarizing the Interpretable Score are applied to facilitate comparison with the simulated disease labels, the top 75th, 80th and 85th percentile of scores are coded as “disease”, **G.** Boxplots depicting the proportion of correct predictions by MiloR. Based on the design of the MiloR log fold change metric, thresholds of 2, 4, and 6 are used to evaluate the predicted disease labels for comparison with simulate labels, **H.** Barplots showing the proportion of correct predictions by CNA. In CNA, cells passing an FDR of 0.05 are considered to be significantly associated, which is used herein to compare with the simulated disease status labels.

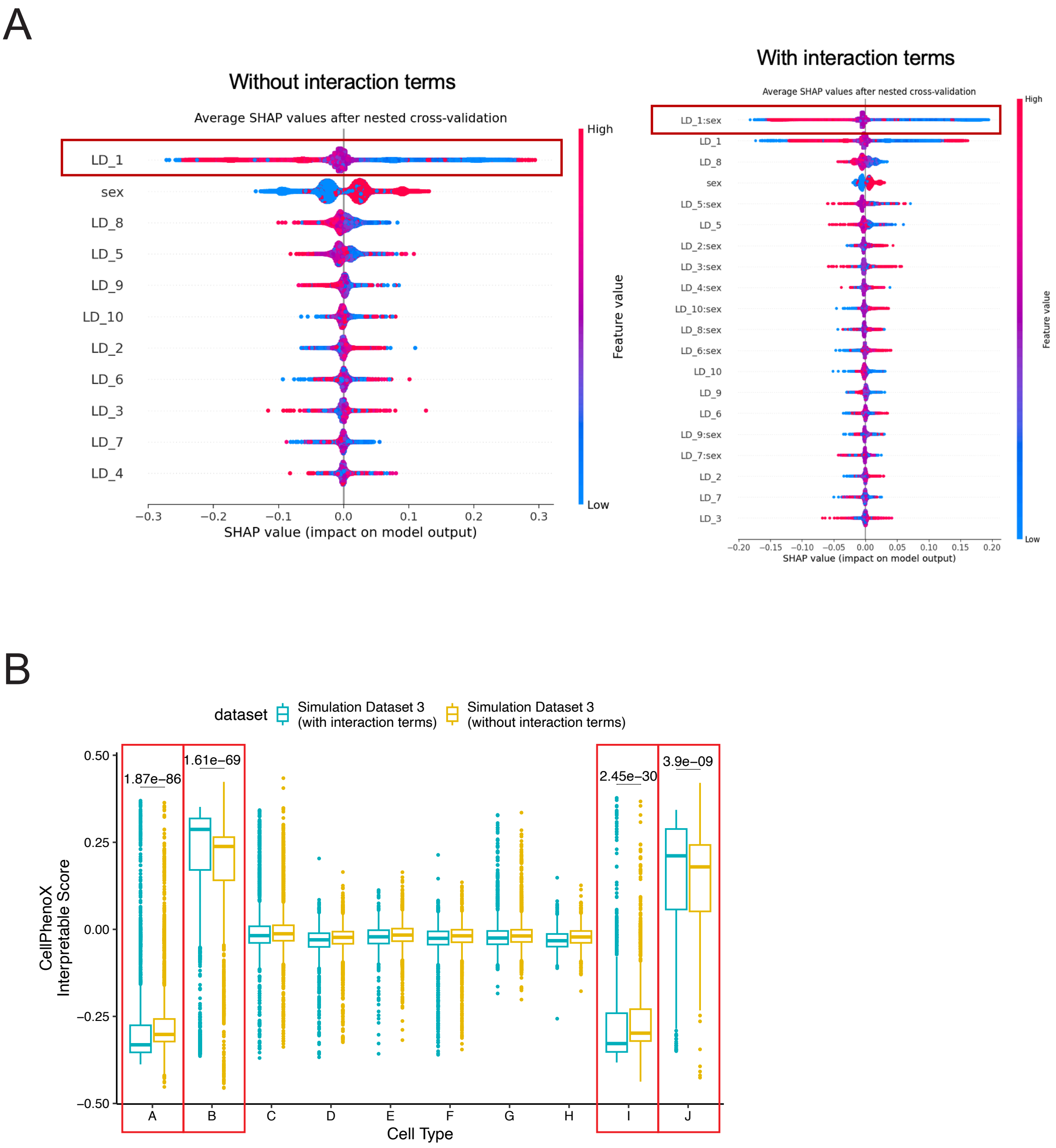

Supplementary Figure 4. SHAP summary of the analysis for simulated data with interaction effects. **A.** SHAP summary plot showing the principal components (PCs) with PC:sex interaction terms (left) and just PCs (right) ranked by mean absolute SHAP value. Each point represents an individual cell, with color indicating the original feature value and position along the x-axis representing the corresponding SHAP value. **B.** Boxplots showing the interpretable score for cells from Females by cell type and model design (including interaction terms in the model and not including interaction terms), Bonferroni adjusted p values reported based on Wilcoxon Test.

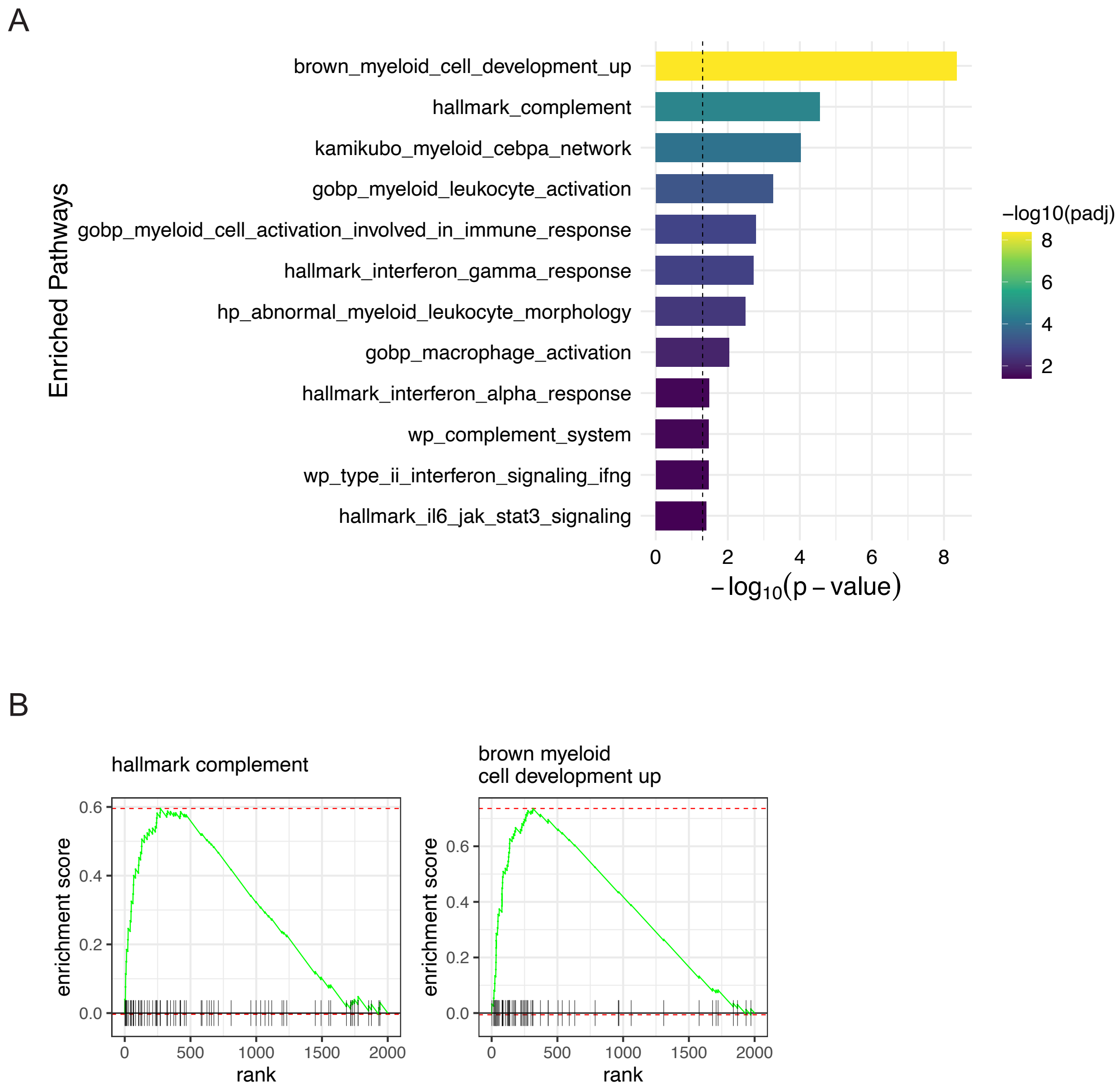

Supplementary Figure 5. Biological validation of CellPhenoX interpretable score using gene set enrichment analysis for the COVID-19 data analysis. A. Barplots depicting enriched pathways based on the correlation between gene expression and the interpretable score. **B.** Enrichment score plots for the top two pathways: Hallmark complement, and Brown myeloid cell development.

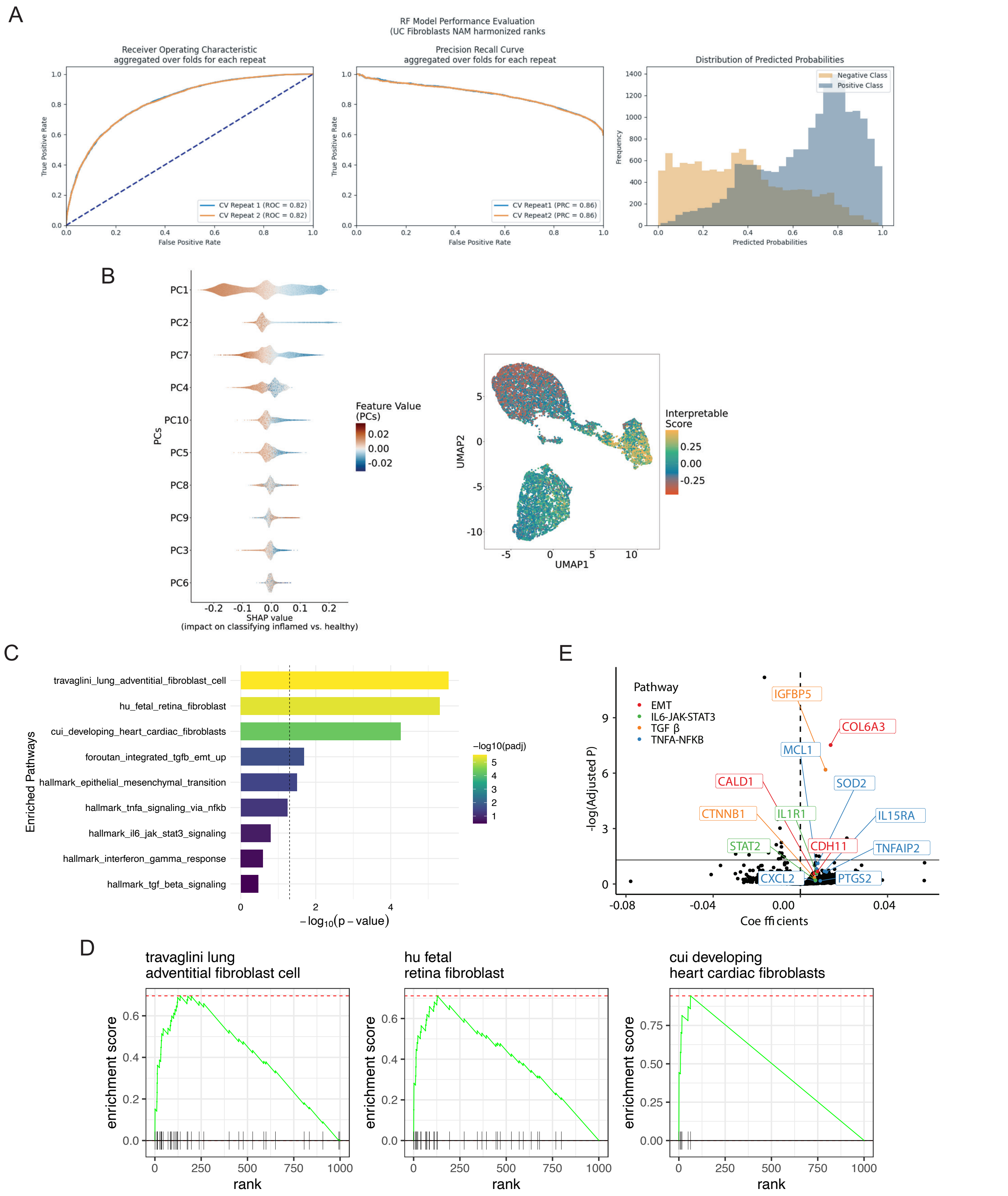

Supplementary Figure 6. Further analytical details of the single-cell ulcerative colitis dataset. A. SHAP summary plot showing the principal components (PCs) ranked by mean absolute SHAP value, where each point represents an individual cell, the color denotes the feature value, and the position along the x-axis represents the corresponding SHAP value (left); UMAP plot colored by the CellPhenoX Interpretable Score (right). **B.** Random Forest performance for the NMF model described in Figure 4, including the AUROC curves for the different CV repeats (left), the AUPRC curves (middle), and histograms showing the predicted probabilities for the positive (inflamed) and negative (non-inflamed) classes. **C.** Barplots showing significantly enriched pathways from gene set enrichment analysis based on the correlation between our interpretable score and gene expression. **D.** Enrichment score plots for the top three pathways: Travaglini lung adventitial fibroblast cell, Hu fetal retina fibroblast, and Cui developing heart cardiac fibroblasts. **E.** Volcano plot of differential gene expression analysis, with colored points highlighting genes from key pathways, including TGFB and epithelial mesenchymal transition (EMT), Hallmark IL6 JAK STAT3 signaling (IL6-JAK-STAT3), Hallmark TGFb signaling, and Hallmark TNFa-NFkB pathways.

Supplementary Figure 7

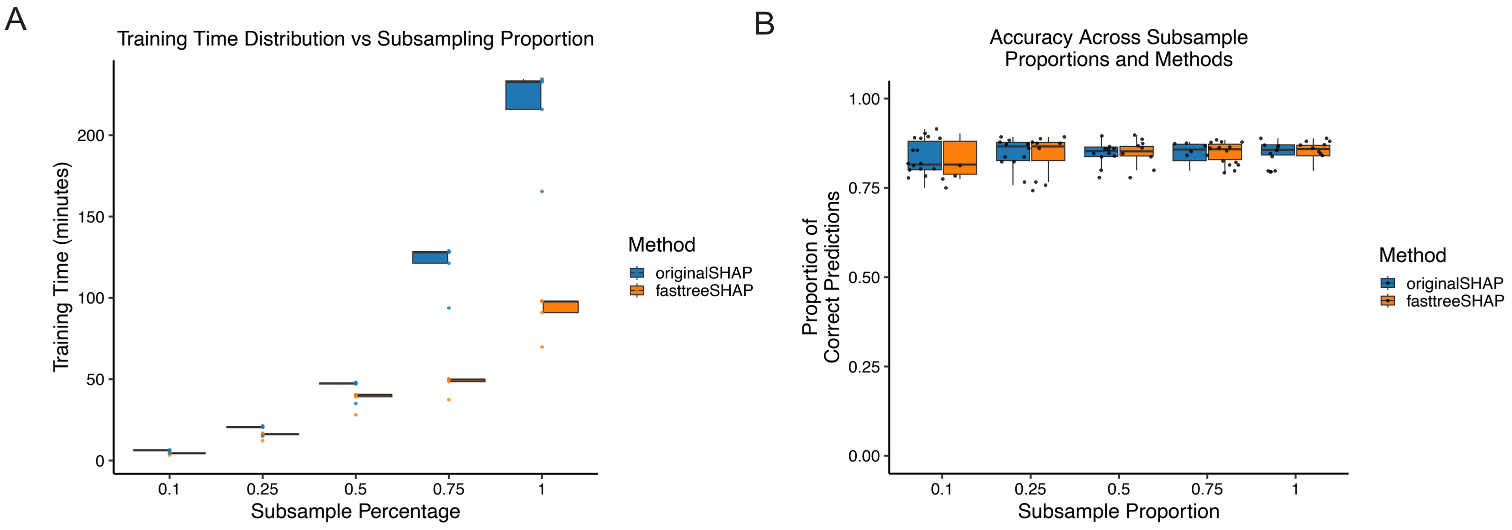

Supplementary Figure 7. Time efficiency and accuracy of Fast TreeSHAP. A. Time efficiency. The average time for Fast TreeSHAP and SHAP method is displayed in the boxplot with cell number changing ranging from 4,102, 10,256, 20,524, 30,784, and 41,040 with stratified downsampling proportion on Simulation Dataset 1. **B.** Accuracy of predictions. The proportion of correct predictions at differing downsampling proportions are shown, where dots represent the accuracy for individual cell types.
